# How AI can learn from the law: putting humans in the loop only on appeal

**DOI:** 10.1038/s41746-023-00906-8

**Published:** 2023-08-25

**Authors:** I. Glenn Cohen, Boris Babic, Sara Gerke, Qiong Xia, Theodoros Evgeniou, Klaus Wertenbroch

**Affiliations:** 1 The Petrie-Flom Center for Health Law Policy, Biotechnology, and Bioethics at Harvard Law School, The Project on Precision Medicine, Artificial Intelligence, and the Law (PMAIL), Cambridge, MA USA; 2 Harvard Law School, Cambridge, MA USA; 3 University of Toronto, Toronto, ON Canada; 4 Penn State Dickinson Law, Carlisle, PA USA; 5 INSEAD, Fontainebleau, France; 6 INSEAD, Singapore, Singapore

**Keywords:** Health policy, Law, Business

## Abstract

While the literature on putting a “human in the loop” in artificial intelligence (AI) and machine learning (ML) has grown significantly, limited attention has been paid to how human expertise ought to be combined with AI/ML judgments. This design question arises because of the ubiquity and quantity of algorithmic decisions being made today in the face of widespread public reluctance to forgo human expert judgment. To resolve this conflict, we propose that human expert judges be included via appeals processes for review of algorithmic decisions. Thus, the human intervenes only in a limited number of cases and only after an initial AI/ML judgment has been made. Based on an analogy with appellate processes in judiciary decision-making, we argue that this is, in many respects, a more efficient way to divide the labor between a human and a machine. Human reviewers can add more nuanced clinical, moral, or legal reasoning, and they can consider case-specific information that is not easily quantified and, as such, not available to the AI/ML at an initial stage. In doing so, the human can serve as a crucial error correction check on the AI/ML, while retaining much of the efficiency of AI/ML’s use in the decision-making process. In this paper, we develop these widely applicable arguments while focusing primarily on examples from the use of AI/ML in medicine, including organ allocation, fertility care, and hospital readmission.

## Introduction

Artificial intelligence (AI) and Machine learning (ML) algorithms—we refer to these as AI/ML—which include a so-called “human-in-the-loop” (HITL) component are increasingly pervasive and sought after by most institutions attempting to partially automate their decision-making processes, including those in medicine, finance, commerce, criminal justice, and other domains where one party (a physician, lender, manager, or judge) makes decisions that affect another (a patient, applicant, customer, or defendant). Whether a lender predicts credit risk, a school predicts student success or a hospital assesses resource needs, a key question is how to best combine humans and AI/ML to maximize the overall quality of the decisions.

We use the phrase HITL very broadly to capture any AI/ML system wherein humans have a role in the ultimate decision-making process. Current HITL discussions focus on involving people to validate an AI/ML’s output and make the final decision. Drawing insights from law and psychological research on judgment and decision-making, we discuss instead how to design a *human-on-appeal* approach that focuses on integrating humans *after* an AI/ML decision and only if requested, based on appropriate appeals processes. Designing a system so that the human judgment enters at the appellate level can, in many cases, leverage the unique strengths of both machine and human judgment while reducing HITL costs and ensuring that those affected by AI/ML decisions are given a voice. We draw analogies to the considerations in designing appeals in court systems to explore how system designers should think about designing appeals processes involving medical decisions made by an AI/ML system. At the same time, our proposal is more general than that as our suggestions can be incorporated into any AI/ML prediction and selection systems in the domains listed above and into any AI/ML dispute resolution scheme that takes place outside the confines of the legal system. For example, companies developing or using AI/ML systems can setup specialized processes and teams to review appeal requests from end users or anyone affected by the AI/ML decisions—similar to customer support but focusing instead on specific AI/ML decisions and not on, say, product repairs or returns. Our main domain of application is medical AI/ML, although our arguments apply across other AI/ML domains, including those listed above.

## The case for human appeals of medical AI/ML decisions

We take as a starting point that many, perhaps most, organizations in healthcare are interested in building AI/ML systems with some HITL component. Accordingly, we provide a framework for thinking about how to structure that HITL component, that is, a framework for where and when in the loop we should place the human, as opposed to whether the human should be in the loop, to begin with. While appeals of AI/ML decisions can be considered in any medical or other context, they may be particularly useful in contexts of scarce resource allocation in which an AI/ML decision affects multiple parties whose incentives are not clearly aligned (e.g., the self-interest of patients versus the economic or communal interest of health insurers, medical providers, or ethics boards) and where there is a risk that the decision may be viewed as unfair^1^. For example, in the context of organ allocation, where those in need of organs far outnumber donors, it would be particularly valuable to have a clear system for appealing an initial AI/ML decision. Other examples of healthcare selection and allocation decisions include the allocation of ventilators, scarce vaccine doses, or hospital beds. Note that the appellant can, in general, be not only a patient but also a family member, a personal doctor, an insurer, or anyone impacted by the AI/ML decision or representing someone impacted. Economists and social scientists often refer to those resources whose use by one person or party precludes the use by another as “rival goods.”^2,3^ Of course, appeals processes may also be desirable in resource allocation decisions where no AI/ML is involved and experts are the only decision-makers, yet we propose that they can be particularly helpful in AI/ML decision-making in areas that are characterized by concerns about fairness^1^, safety^4^, trust, and unique circumstances such as in medical decision-making^5,6^.

While recognizing that context matters and that the best HITL designs will reflect the specifics of particular decision-making domains (e.g., organ donations versus consumer credit decisions), there are several generalizable reasons to involve humans only on appeal rather than involving them to validate every AI/ML decision.

First, involving a human expert only on appeal can significantly decrease the cost and increase the scale of AI-based decision-making—as the expectation would be that appeals will only be requested in some cases, especially if constraints and costs are built into the appeals process design that parallels those in judiciary appeals, which we discuss below. In particular, where the use of human expertise is expensive or in short supply, involving human experts only on appeal can leverage their impact.

Second, appealed cases may draw attention to, and be more informative about, relevant but unincorporated variables, providing for the discovery of important data to improve an AI/ML^7–10^. That is because the person or patient who is impacted by the AI/ML decision may offer unique insights into their case during the appeal that may be overlooked when a HITL validates every AI/ML decision.

Third, appeals provide a greater sense of engagement, trust, and control over the decision-making process, giving a voice to the people impacted. Lack of trust and control is one of the key reasons for algorithm aversion^11^, with users lacking trust in AI/ML decisions particularly when the underlying task domain is subjective, or involves moral or esthetic preferences^12–14^. As an example, consider a fertility use case: the use of AI/ML to automate the embryo ranking and selection procedure by extracting relevant information from embryo microscopy images and then helping a fertility specialist decide which embryo to implant^15^. Patients that are young and/or have sufficient resources may be content with an AI/ML making a final determination of whether the embryo should be implanted since the patients know that if none of the embryos they have produced are deemed appropriate for implantation by the AI/ML they may begin another in vitro fertilization (IVF) cycle and try again. By contrast, when the patients are older (in particular women who may not have many eggs left to retrieve) or lack resources for future IVF cycles, they may have strong preferences to have the option to “appeal” whatever the AI/ML recommends to the embryologist, since accepting the AI/ML decision may mean they never reproduce at all.

Fourth, including a human during the initial decision-making stage can often make decisions worse, for example, in unfamiliar task environments^8^, by considering potentially biased information about the object^16,17^, and because human judges are less reliable than algorithms^18,19^.

Fifth, moral considerations can also be evaluated more carefully at the appeal stage, which enables organizations to avoid the pitfalls of trying to “automate morality” or of creating a system that is unduly technocratic^20,21^.

Finally, by placing the human at the appeal stage, we allow for the correction of AI/ML errors and the addition of relevant information that may be idiosyncratic, without always having a HITL. For example, while an algorithm that helps determine prioritization for kidney transplantation may appropriately consider the distance between the deceased donor and the recipient and its effects on waiting time, a human reviewer at the appellate stage can identify the exceptional case. For instance, Hawaii is so far from the continental United States that the travel and thus waiting time for an organ will be unusually long, and a reviewer on appeal might conclude that this justifies an outcome different from the original determination. Psychologists and decision researchers refer to such cues that are highly influential or diagnostic yet occur with such rarity that they are not included in a statistical prediction algorithm (e.g., for organ allocation) as “broken-leg cues” (e.g., a recently broken leg prevents a professor from going to the movies, despite an algorithmic prediction that the person will go, an emblematic example described by Paul Meehl)^18,19^.

## Designing appeals for medical AI/ML decisions: lessons from the judiciary system

While HITLs may potentially offer all these benefits at the appeal stage, realizing them depends on the appropriate appellate design. We derive lessons for how to design appeals of AI/ML decisions by looking at the way court systems design appellate procedures. Research in social psychology has long suggested that offering options to appeal decisions can enhance perceptions of procedural fairness^22^. Thus far, however, there is little direct empirical evidence comparing different kinds of HITL systems (but see ref. ^23^ for a test of allowing users to appeal to an internal review board), and collecting such evidence would be challenging as such systems are in their nascency. That is just the kind of research we hope to encourage with this proposal.

To use the example of one legal jurisdiction (one would find parallels across the world), the most basic description of the U.S. federal court system’s appellate process is as follows: A district court acts as the trial court with a single judge (sometimes supplemented by jurors) making the key decisions. The losing party can take an appeal to a three-judge “Circuit Court,” and the losing party on appeal may seek discretionary review in the nine-Justice U.S. Supreme Court, which determines (in most instances) what cases to take. Several features of appellate review could be used as design choices for optimally integrating a human in the loop on appeal. We focus on three such features but stress that we are simply using appeal to a court as an analogy—AI decisions could instead be appealed to an organization or team that developed or deployed the AI/ML that makes these decisions. We understand HITL on appeal as being part of an organization’s own ongoing risk management structure, the kind of controls that happen before in-house counsel is involved or legal proceedings are initiated. These processes could also be more formally structured while remaining outside the formal legal system as a form of alternative dispute resolution. For example, several (proposed) regulations for online trust and safety, such as the EU’s Digital Services Act^24^, require that online platforms put in place out-of-court dispute settlement mechanisms when a user and a platform disagree about the removal of the user’s content (or of the user’s account).

### Standard of review

In court systems, not all issues are reviewed under the same standard on appeal: For example, some issues are reviewed “de novo” without any deference to the lower court decisions, while others only for “clear error” with substantial deference to the trial court. These standards are typically set with the epistemic and other advantages of the decision-makers in mind. For example, the trial court judge sees the testimony of witnesses and can observe their behavior, while the appellate court is particularly suitable for resolving “pure questions of law.”

In designing appeals of AI/ML decisions, we should likewise consider the standard of review by reference to the epistemic and other advantages and disadvantages of humans versus AI. For example, if the question is purely about accuracy—such as determining whether a spot in a particular area on an X-ray should be flagged for further review—AI may be superior, and cases may be reviewed only for “clear error” without necessarily changing the AI/ML model beyond possibly retraining it. By contrast, if the question is about whether an AI/ML model might be misspecified or unfair, human decision-makers may be well poised to consider the full range of relevant considerations, including moral/legal ones, and “de novo” review of the overall AI/ML can be considered. For example, Obermeyer et al.^25^ showed how a hospital readmission algorithm that uses health costs as a proxy for health needs produced racially biased results. Because black patients tend to be less costly in their treatment in the data set, the algorithm recommends fewer resources for them even at the same level of need. This is the type of error that human reviewers may *more* easily spot (though to be sure such “label bias” is not that easily detected).

### Centralization of review and consolidation of cases for more expertise

In most civil and criminal cases in the U.S. federal courts, a review of a trial court decision in one of the 94 district courts goes to one of the 12 U.S. Courts of Appeals (“Circuit Courts”) that are geographically coincident with the district court in question. In some areas, though, the system designers have elected to channel all appeals to a single Circuit Court. The most prominent example is patent decisions, which go to a specialized court whose judges tend to have more scientific background and patent law experience than the average Circuit Court judge.

We can imagine similar federated structures for AI/ML appeals. For example, when the same type of AI/ML algorithm or data is used, or for similar AI/ML applications, the issue may be directed to a centralized specialized team in an organization, whose members would become increasingly sophisticated in their review of particular issues. They might also be able to develop more general principles on topics like tradeoffs between AI/ML accuracy and explainability or on AI/ML fairness, which would not only be “adjudicative” of particular cases but could generate norms for AI/ML developers, much like precedent operates in the court system. Research ethics provides some interesting analogies here: Institutional Review Boards (called Research Ethics Boards in some countries or other names) often develop sophistication when they review multiple studies in the same area (for example, HIV drugs or adolescent populations). They also often informally develop systems of “precedents,” where prior decisions on similar issues are used as a basis for new variations. Another analogy is to a “tumor board,” which brings together cancer and other doctors at a hospital system to decide as a group on the best treatment plan for a patient in complex cancer cases.

A different form of centralization to consider is at the level of cases, not courts. In the U.S. Federal courts, it is sometimes possible to consolidate a series of separate cases (for example, claims that a rent-a-car company added hidden fees to many customers) at the trial level through a class action. It is also possible to consolidate several cases together for the appeals stage, even if they were tried separately. In the AI/ML context, this possibility might be of particular value—there may be issues with the actual deployment of an algorithm that only become manifest when one looks at a large number of errors. The hospital readmission algorithm example above illustrates this. In any particular case, the nature of this problem may not be clear, but if enough are seen collectively on appeal, the bias becomes manifest.

There is a myriad of possible design choices that this opens up for consideration depending on context. When the decision in question is by an AI/ML system designed and applied in a hospital system to a particular clinical case, it would seem intuitive for the reviewing body on appeal to be situated in that particular hospital system. But if the same AI/ML is used in many different hospital systems across the country one might imagine a better design might be a centralized appellate review body—potentially a regulated third-party entity, similar, for example, to the role of accounting auditors—to which all the hospitals feed cases for review. Whether that approach is plausible may depend on how much the AI/ML has to be calibrated to the hospital or health system it is used in as well as orthogonal legal questions such as whether such an outside review body would increase a hospital system’s liability exposure, the permissibility of sharing patient data with others, etc. An in-between option might be to develop “template” appeals processes that could be implemented at each hospital system that uses the AI/ML—this would keep review “in house” but provide some consistency and also allow some gathering of information from each hospital regarding what happens in their appeals. We believe human-on-appeal designs can provide value irrespective of whether the particular medical AI/ML requires review by a regulator like the U.S. Food and Drug Administration (FDA) or not. What matters is the type of decision and the stakes involved which does not perfectly track the regulator’s jurisdiction in the U.S. or in other countries. That said, for the subset of medical AI/ML that does require review by regulators like the FDA, regulators may be able to consider, encourage, or even require some forms of appeal design as part of their regulatory review.

### Glide paths or obstacle courses?

Another lesson from the U.S. court system is that the mere existence of an appellate review process does not guarantee it will be used, let alone in an optimal way. This provides one of the most important lessons for appeals of AI/ML decisions. If every AI/ML decision is automatically reviewed by human judges—which is effectively how many current HITL approaches typically operate, where the human reviews every prediction by default—we may lose some of the advantages of automation in the first place. This suggests a design where affected parties do not have an automatic right to appeal (as with the U.S. Circuit courts) but grants the appellate panel discretion on whether to grant a review or not (as with the U.S. Supreme Court’s certiorari power to control which cases it hears for the most part). We might also consider imposing penalties for frivolous appeals, imposing a flat fee to take an appeal, or imposing a cost automatically on the losing party. Meanwhile, if we worry that only sophisticated or well-resourced parties will take appeals even when there is merit to them, we might think of building a set of free “appeal assistants” in analogy to the public defender in criminal law or legal aid societies in the civil system. For medical AI, such assistance may be particularly important for poor or otherwise vulnerable populations who may find it more difficult to challenge decision-making presented with the air of authority. One might even consider providing a monetary incentive to take an appeal provided one wins; a loose analogy is to *qui tam* actions under the False Claims Act, where the one who refers the case to the government gets a share of the ultimate sum won by the government. The final design choices will depend on whether the designers of the appeals process think those deciding whether to take an appeal are in a good epistemic position to know the merits of the case or not—or on their technical literacy to be able to understand the details of the specific AI.

## Best of both worlds

Current debates about AI/ML versus humans or about a HITL limit the possibilities of how to best combine AI/ML and people, leading to tradeoffs such as those between accuracy and trust. However, we may be able to get the best of both worlds—an accurate, efficient, and trustworthy combination of human expertise and AI—if we design proper human appeals of AI/ML decisions. How these processes should be designed and where a human should be placed can be context-specific—and an area of future research. We propose that deploying HITL on appeal, modeled on the design of appeals processes in the legal domain, may fruitfully expand debates about HITL.

## Data Availability

This article does not make direct use of any data.
